# Prevalence and Antimicrobial Resistance Profiles of *Salmonella* Isolated From Table Eggs in Rupandehi, Nepal

**DOI:** 10.1002/vms3.71052

**Published:** 2026-07-25

**Authors:** Maadhabi Pokhrel, Akash Adhikari, Sujan Adhikari, Priyanka Bhatta, Samikshya Bhusal, Swagat Khanal, Abhishek Adhikari, Suman Kumar Singh, Birendra Shrestha

**Affiliations:** ^1^ Institute of Agriculture and Animal Science (IAAS) Tribhuvan University, Paklihawa Campus Bhairahawa Nepal; ^2^ Faculty of Animal Science, Veterinary Science and Fisheries Agriculture and Forestry University Rampur Chitwan Nepal; ^3^ Department of Veterinary Surgery, Medicine, Epidemiology and Public Health Institute of Agriculture and Animal Science (IAAS) Tribhuvan University, Paklihawa Campus Bhairahawa Nepal; ^4^ Department of Veterinary Epidemiology and Public Health Institute of Agriculture and Animal Science (IAAS) Tribhuvan University, Paklihawa Campus Bhairahawa Nepal

**Keywords:** AMR, Nepal, *Salmonella*, table eggs

## Abstract

**Background:**

Non‐typhoidal *Salmonella* (NTS) is a leading cause of foodborne illness globally, with poultry eggs serving as a primary vehicle for human infection. The emergence of antimicrobial resistance (AMR) in *Salmonella*, particularly to critically important antibiotics, compounds this public health threat.

**Objectives:**

This cross‐sectional study, conducted in the Rupandehi district of Nepal, aimed to assess *Salmonella* prevalence in table eggs and characterize the AMR profiles of recovered isolates.

**Methods:**

A total of 150 eggs were collected from layer farms (*n = *45), retail shops (*n = *60) and restaurants (*n = *45). *Salmonella* was isolated using standard culture‐based methods, and antimicrobial susceptibility was determined via the Kirby–Bauer disk diffusion method.

**Results:**

The overall prevalence of *Salmonella* was 10.0% (95% CI: 5.6%–16.2%). A statistically significant contamination gradient was observed across the supply chain, with prevalence increasing from 2.2% at farms to 15.0% at retail shops and 11.1% at restaurants (Fisher's exact test, *p *< 0.05). Logistic regression analysis identified retail shops as a significant risk factor for contamination compared to farms (OR = 7.6). Antimicrobial susceptibility testing of 17 isolates revealed alarmingly high resistance rates to ciprofloxacin (88.2%), tetracycline (76.5%) and ampicillin (70.6%). Furthermore, 58.8% of isolates were multidrug‐resistant (MDR).

**Conclusions:**

These findings reveal systemic failures in post‐farmgate hygiene and underscore the urgent need for integrated One Health interventions, including enhanced surveillance, improved food handling practices and stringent antimicrobial stewardship in the poultry sector to mitigate this pressing public health risk.

## Graphical abstract

This study found a 10.0% overall prevalence of *Salmonella* in table eggs in Rupandehi, Nepal, with contamination increasing significantly from farms to retail outlets. The isolates exhibited alarming multidrug resistance (58.8%), including 88.2% resistance to ciprofloxacin, underscoring severe post‐farm hygiene failures and a critical One Health threat.

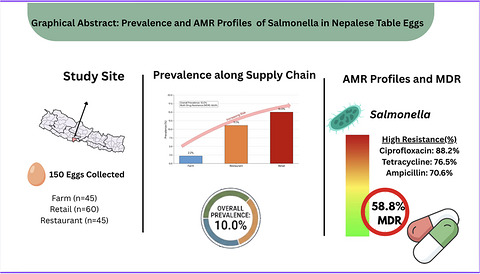

## 1 Introduction

Non‐typhoidal *Salmonella* (NTS) is predominantly foodborne and zoonotic, with transmission linked to contaminated animal products, fresh produce and water sources (Dudhane et al. 2023; Sodagari et al. 2020). The burden is concentrated in low‐ and middle‐income countries, where weak sanitation, unsafe water and limited food‐safety infrastructure facilitate pathogen transmission (Lamichhane et al. 2024; Subedi et al. 2025). Clinical outcomes range from self‐limiting enteritis to invasive disease that requires parenteral therapy, with the highest risk borne by children under five, the elderly and immunocompromised persons (Berkley et al. 2005; Nazir et al. 2025). Considerable economic costs are imposed through healthcare spending, lost productivity and disability‐adjusted life years (Torres et al. 2024).

Poultry and poultry‐derived products are commonly implicated as reservoirs and transmission vehicles for NTS. Chickens frequently carry *Salmonella* asymptomatically in the intestinal tract, enabling entry of the pathogen into the food chain (Shaji et al. 2023). Egg contamination is mediated by two principal routes: vertical (trans‐ovarian) transmission occurs when the hen's reproductive tract is infected and the egg contents are contaminated prior to shell formation, whereas horizontal transmission arises when eggshells are exposed to faecal or environmental contamination after laying (Pande et al. 2016). Both routes increase the capacity of eggs to transmit *Salmonella* and to trigger outbreaks. This leads to economic impacts on the poultry sector including production losses, recalls and market disruption (Kulshreshtha et al. 2022; Samiullah et al. 2013).

The problem of egg‐associated *Salmonella* is intensified by antimicrobial resistance (AMR). Routine antimicrobial use in animal husbandry for treatment, prevention and growth promotion creates selective pressure that favours resistant strains (Punchihewage‐Don et al. 2022). Mobile genetic elements facilitate the transfer of resistance determinants across bacterial populations, accelerating the dissemination of AMR in poultry reservoirs (Partridge et al. 2018). South Asia has been identified as a hotspot for AMR emergence, where rapid poultry‐sector intensification has often outpaced improvements in biosecurity and regulatory oversight (Abreu et al. 2023; Acharya et al. 2023). The misuse of antimicrobials establishes a feedback loop in which production demands drive drug administration, selecting for resistant pathogens that subsequently enter the food supply, ultimately increasing the human clinical burden (Oliveira et al. 2024).

Nepal exemplifies these converging risks. Rapid expansion of the poultry sector has contributed to food security and livelihoods, but high AMR prevalence in poultry products and limited stewardship have been reported (S. Sharma et al. 2021). Systematic data on *Salmonella* contamination of table eggs were scarce for many districts, including Rupandehi, an important agricultural and commercial hub. This study was therefore undertaken to estimate *Salmonella* prevalence in eggs from layer farms, retail outlets and restaurants in Rupandehi and to characterize the antimicrobial susceptibility profiles of recovered isolates against clinically relevant agents. The aim was to inform targeted, evidence‐based public health interventions.

## 2 Materials and Methods

### 2.1 Study Design and Sample Collection

**FIGURE 1:**
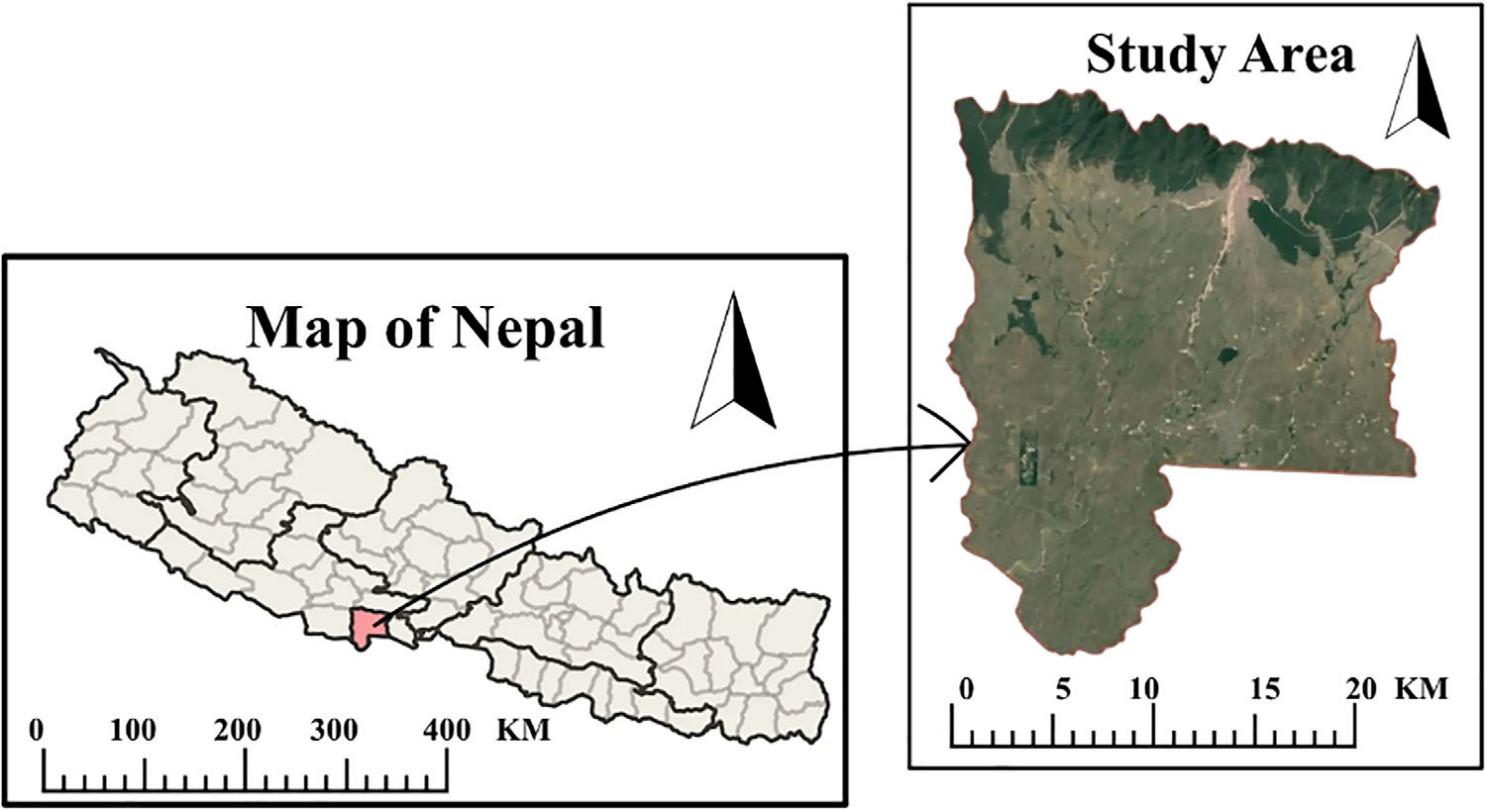
Map of Nepal indicating the study area.

A cross‐sectional study was carried out in the Rupandehi district of Nepal (Figure 1) from August to October 2023. Stratified purposive sampling was applied to capture potential contamination across key stages of the egg supply chain, from production to consumption (Patton 2002). A total of 150 eggs were collected, comprising 45 from commercial layer farms, 60 from retail shops and 45 from restaurants. Each sample was handled aseptically, sealed in sterile zipper bags and transported in a cold box to the laboratory.

### 2.2 *Salmonella* Isolation and Identification

Eggshells and contents were processed separately to assess external and internal contamination. Eggshells were aseptically crushed, and 1 g of material was suspended in 9 mL of buffered peptone water (M1494I, HiMedia, India) for pre‐enrichment, while 1 mL of homogenized egg contents was added to 9 mL of BPW. This crushing technique was employed to ensure the recovery of *Salmonella* localized both on the surface and within the shell pores, a method considered superior to swabbing as it captures bacteria that may have penetrated the shell matrix or adhered to the inner membrane (Musgrove et al. 2005).

All suspensions were incubated at 37°C for 24 h. For selective enrichment, 1 mL of each pre‐enriched culture was transferred to 5 mL of Selenite Cystine broth (M025, HiMedia, India) and incubated at 37°C for 4–6 h. A loopful of the enriched culture was then streaked onto xylose–lysine–desoxycholate agar (M031, HiMedia, India) and *Salmonella*–*Shigella* agar (M108, HiMedia, India), both incubated at 37°C for 24 h. Colonies with typical *Salmonella* morphology, such as pink centres with black cores, were recorded as presumptive positives. Presumptive isolates were confirmed using a standard biochemical panel, including Gram staining, catalase, oxidase, motility, indole, methyl red, Voges–Proskauer and citrate utilization tests.

### 2.3 Antimicrobial Susceptibility Testing

Antimicrobial susceptibility testing (AST) was performed on all confirmed isolates by the Kirby–Bauer disk diffusion method on Mueller–Hinton agar, following CLSI guidance (CLSI 2024). Well‐isolated colonies were suspended in nutrient broth and adjusted to a 0.5 McFarland turbidity standard. Plates were inoculated using a sterile swab to ensure even coverage.

Seven antibiotic disks representing different classes were tested (Table 1). Plates were incubated aerobically at 37°C for 24 h. Zone diameters were measured in millimetres and interpreted as susceptible, intermediate, or resistant according to CLSI breakpoints. Isolates showing acquired non‐susceptibility to at least one agent in three or more antimicrobial classes were classified as multidrug‐resistant (MDR) (CLSI 2024).

**TABLE 1:** Panel of selected antibiotic disks and potencies utilized in disk diffusion assay.

Antibiotic name	Disk potency
Ciprofloxacin (CIP)	5 µg
Tetracycline (TET)	30 µg
Ampicillin (AMP)	10 µg
Trimethoprim–sulfamethoxazole (SXT)	25 µg
Chloramphenicol (CHL)	30 µg
Ceftriaxone (CTR)	30 µg
Amikacin (AMK)	30 µg

### 2.4 Statistical Analysis

Data were entered into Microsoft Excel and analysed in IBM SPSS Statistics v25. Prevalence estimates were calculated as proportions. Exact 95% confidence intervals were computed using the Clopper–Pearson method. The association between sampling source (farm, retail and restaurant) and *Salmonella* prevalence was tested using Fisher's exact test, chosen because expected counts in some farm cells were < 5. A two‐sided *p*‐value < 0.05 was considered statistically significant.

To quantify differences across the supply chain, a multivariable logistic regression model was fitted. The binary outcome was *Salmonella* contamination (1 = positive, 0 = negative). Sampling source was the primary independent variable, while simultaneously adjusting for potential confounders, specifically the sample component type and temporal factors corresponding to the month of collection. Adjusted odds ratios and their 95% confidence intervals were reported to estimate the relative odds of contamination at retail and restaurant points compared with farms (Hosmer and Lemeshow 2000). A geographic map of the study area was generated using QGIS software (LTR 3.44) (Figure 1).

## 3 Results

### 3.1 Prevalence of *Salmonella* Contamination

Of 150 table eggs examined, 15 were positive for *Salmonella*, giving an overall prevalence of 10.0% (95% CI: 5.6%–16.2%). Contamination was detected more frequently on eggshells (13/150; 8.7%; 95% CI: 4.6%–14.6%) than in egg contents (4/150; 2.7%; 95% CI: 0.7%–6.7%). Two eggs were positive in both shell and content.

### 3.2 Supply‐Chain Contamination Gradient and Statistical Significance

A clear gradient of contamination was observed along the supply chain. Eggs collected at the farm gate had the lowest prevalence (1/45; 2.2%; 95% CI: 0.1%–11.7%). Prevalence increased at consumer‐facing points: 9/60 (15.0%; 95% CI: 7.1%–26.6%) among retail shop samples and 5/45 (11.1%; 95% CI: 3.7%–24.1%) among restaurant samples. The difference in prevalence across the three strata was statistically significant (Fisher's exact test, *p* = 0.048), indicating that contamination levels increased from farm to downstream nodes in the supply chain (Table 2).

**TABLE 2:** Prevalence of *Salmonella* in table eggs by source and component (*n* = 150).

Point of collection	No. samples	No. (%) positive (overall)	95% CI	No. (%) positive (shell)	No. (%) positive (content)
Layer farms	45	1 (2.2%)	0.1%–11.7%	1 (2.2%)	0 (0.0%)
Retail shops	60	9 (15.0%)	7.1%–26.6%	8 (13.3%)	3 (5.0%)
Restaurants	45	5 (11.1%)	3.7%–24.1%	4 (8.9%)	1 (2.2%)
Total	150	15 (10.0%)	5.6%–16.2%	13 (8.7%)	4 (2.7%)

*Note*: Shell and content positives are not mutually exclusive; two eggs were positive in both components.

### 3.3 Risk Factors for Contamination

Multivariable logistic regression was used to estimate the odds of contamination at post‐farmgate points relative to farm‐sourced eggs. After adjusting for sample type and temporal factors, eggs from retail shops exhibited higher odds of contamination compared with farm eggs (OR = 7.60), although the confidence interval was wide (95% CI: 0.92–63.51) and the result was marginally non‐significant (*p* = 0.059). Elevated odds were also observed for restaurant‐sourced eggs (OR = 5.51; 95% CI: 0.61–51.13; *p* = 0.130). The regression results are shown in Table 3.

**TABLE 3:** Multivariable logistic regression for factors associated with *Salmonella* contamination.

Predictor (ref = Farm)	Odds ratio (OR)	95% CI	*p*‐value
Retail shop	7.60	0.92–63.51	0.059
Restaurant	5.51	0.61–51.13	0.130

### 3.4 Antimicrobial Resistance and Multidrug Resistance

Seventeen unique *Salmonella* isolates were recovered from the 15 positive eggs (two eggs yielded both shell and content isolates). All isolates underwent AST. High resistance rates were observed to several clinically important agents: ciprofloxacin 88.2% (15/17), tetracycline 76.5% (13/17), ampicillin 70.6% (12/17), trimethoprim–sulfamethoxazole 64.7% (11/17), chloramphenicol 58.8% (10/17) and ceftriaxone 52.9% (9/17). Amikacin showed the lowest resistance (17.6%; 3/17). Ten isolates (58.8%) met the study definition of multidrug resistance (resistance to ≥ 1 agent in ≥ 3 antimicrobial classes). Detailed resistance phenotypes are presented in Table 4.

**TABLE 4:** Antimicrobial resistance phenotypes of *Salmonella* isolates (*n *= 17).

Antimicrobial class	Agent	No. (%) resistant	No. (%) intermediate	No. (%) susceptible
Quinolones	CIP	15 (88.2%)	0 (0.0%)	2 (11.8%)
Tetracyclines	TET	13 (76.5%)	1 (5.9%)	3 (17.6%)
Penicillins	AMP	12 (70.6%)	2 (11.8%)	3 (17.6%)
Folate pathway antagonists	SXT	11 (64.7%)	3 (17.6%)	3 (17.6%)
Phenicols	CHL	10 (58.8%)	4 (23.5%)	3 (17.6%)
Cephalosporins (3rd gen)	CTR	9 (52.9%)	4 (23.5%)	4 (23.5%)
Aminoglycosides	AMK	3 (17.6%)	5 (29.4%)	9 (52.9%)

## 4 Discussion

This study provides the first systematic investigation of *Salmonella* contamination in commercial table eggs from Nepal's Rupandehi district. Among 150 eggs sampled across farms, retail shops and restaurants, the overall prevalence of *Salmonella* was 10.0%. This level is comparable to other reports from South Asia and underscores the substantial burden of egg‐associated *Salmonella* in the local food system (Solís et al. 2023). Contamination occurred more frequently on eggshells (8.7%) than in egg contents (2.7%), indicating that horizontal transmission is the primary route of contamination. Faecal or environmental contamination of eggshells after laying appears to be the dominant mechanism. Nevertheless, the detection of *Salmonella* inside eggs, although less common, confirms vertical transmission via infected hens (Liu et al. 2023). This finding suggests lapses in farm biosecurity and hen's health, as some hens must have been laying eggs with ovarian contamination prior to shell formation.

A striking pattern emerged along the supply chain. Eggs collected directly from farms had the lowest prevalence at 2.2%, whereas retail eggs showed a prevalence of 15.0% and restaurant eggs exhibited an intermediate prevalence of 11.1%. Statistical analysis confirmed that the risk of contamination increased downstream (Fisher's exact test, *p* ≈ 0.048). Multivariable logistic regression suggested that retail‐sourced eggs had roughly seven times higher odds of being contaminated than farm eggs, while restaurant‐sourced eggs also displayed elevated risk. These trends, although accompanied by wide confidence intervals, are biologically and epidemiologically significant, highlighting the post‐farm environment as an important amplifier of risk (Nakagawa and Cuthill 2007).

The high prevalence observed at retail shops (15.0%) is likely driven by systemic hygiene failures in the supply chain. A major contributing factor is the reliance on reusable transport crates and containers that are frequently inadequately cleaned, facilitating bacterial transfer between successive batches of eggs. The absence of a consistent cold chain allows *Salmonella* on eggshells to survive or even multiply during transport and storage (Gast et al. 2018). Additionally, cross‐contamination occurs frequently at retail and restaurant points. Limited hygiene practices, including unwashed hands, shared utensils and inadequate cleaning of surfaces, provide opportunities for *Salmonella* to spread from other foods or the environment to eggs (Ehuwa et al. 2021; Foods 2019). Collectively, these findings indicate that systemic failures beyond the farm are driving contamination. While on‐farm biosecurity remains fundamental, targeted interventions in transport, storage and food handling are urgently required to prevent downstream amplification of risk. Simple measures such as routine cleaning of crates, temperature management and hygiene training for handlers could substantially reduce the overall prevalence of contamination (Arjmand et al. 2025; Youssef et al. 2021).

### 4.1 AMR and Public Health Implications

From a clinical perspective, the antimicrobial resistance patterns observed in *Salmonella* isolates are concerning. Among the 17 isolates recovered, 88.2% were resistant to ciprofloxacin, a fluoroquinolone classified as critically important for human medicine and a first‐line therapy for invasive nontyphoidal salmonellosis. This level of resistance implies that standard treatment for infections acquired from eggs may be ineffective, potentially resulting in prolonged illness, complications, longer hospital stays and higher mortality (Dadgostar 2019). Resistance to other antibiotics was also high, with 76.5% resistant to tetracycline, 70.6% to ampicillin, 64.7% to trimethoprim–sulfamethoxazole, 58.8% to chloramphenicol and 52.9% to ceftriaxone. Only amikacin remained largely effective, with 17.6% resistance. Ten isolates (58.8%) were MDR, defined as resistance to at least one agent in three or more antimicrobial classes. These resistance patterns reflect intense antimicrobial selection pressure within the local poultry industry. The high prevalence of resistance to older drugs, such as tetracycline and ampicillin, is expected in regions with widespread antimicrobial use (Muteeb et al. 2023). However, near‐universal ciprofloxacin resistance suggests indiscriminate use of fluoroquinolones, often without veterinary oversight (Shariati et al. 2022). Self‐medication of flocks and unregulated antibiotic sales are well documented in Nepal and contribute to this problem (Acharya and Wilson 2019; Rijal et al. 2021). Such practices compromise the effectiveness of critical antimicrobials for both animal and human health.

The findings carry clear implications for veterinary regulation and poultry management. Strengthening prescription‐only policies for critical antibiotics is essential. Farmers should consult veterinarians before administering antimicrobials, and extension services should provide accessible guidance on responsible usage. Formal food safety systems, such as hazard analysis and critical control point frameworks, are recommended for poultry operations (G. Sharma et al. 2024). These systems should incorporate routine sanitation of laying areas and transport equipment, temperature control throughout storage and distribution, batch traceability and training of handlers in safe egg handling practices (Sawadogo et al. 2023). Together, these measures can reduce both contamination and the spread of MDR *Salmonella* along the supply chain.

While our study focuses on the contamination of table eggs, the high levels of resistance, particularly the 88.2% resistance rate to ciprofloxacin reflects a significant public health risk in the Rupandehi region. Although our data do not include direct isolates from human clinical cases, the resistance patterns observed in these egg‐derived *Salmonella* isolates mirror global and regional trends where MDR NTS increasingly compromises human treatment outcomes (Dudhane et al. 2023; Lamichhane et al. 2024). The presence of these resistant pathogens in a widely consumed food source suggests a direct, though here unquantified, link to the local burden of difficult to treat foodborne illnesses. This highlights the necessity for future One Health collaborations that integrate veterinary surveillance with human clinical data to fully map the transmission dynamics and clinical impact of resistant *Salmonella* in Nepal.

Consumer education remains a critical component of mitigation strategies. Public awareness campaigns emphasizing proper egg handling, storage and cooking can reduce risk. Key recommendations include inspecting eggs before purchase, avoiding soiled or cracked shells, refrigerating eggs promptly and cooking until yolks are firm, particularly for vulnerable populations (Kosa et al. 2015; Sawadogo et al. 2023). Training for food handlers in markets and restaurants should focus on preventing cross‐contamination through proper hygiene and segregation of raw and cooked foods (Putri and Susanna 2021). These measures complement upstream interventions and form an essential barrier to infection.

## 5 Limitations of the Study and Future Directions

Although this study provides a comprehensive snapshot of *Salmonella* contamination and antimicrobial resistance risks in Rupandehi, it is subject to several limitations. The cross‐sectional design and purposive sampling may not account for seasonal variations or be fully representative of other regions in Nepal. The lack of molecular subtyping and the absence of direct correlation with local human clinical isolates prevent a full assessment of the specific contribution of these eggs to the regional human illness burden, a key requirement for a complete One Health assessment. Standard quality control reference strains were unavailable due to resource constraints. However, to maximize testing accuracy, all disk‐diffusion parameters were strictly standardized and executed in precise accordance with CLSI methodological protocol. The relatively small sample size and number of recovered isolates also resulted in wide confidence intervals in the logistic regression analysis, which may obscure more nuanced risk factors associated with retail and restaurant environments.

To address these gaps, future research should integrate molecular epidemiology, specifically whole‐genome sequencing, to identify circulating *Salmonella* serovars and detect mobile genetic elements responsible for resistance. Such data would allow for the high‐resolution tracing of strains along the supply chain and the identification of specific points of amplification. Incorporating even periodic sequencing could transition routine surveillance into a predictive tool, enabling rapid responses to emerging strains and guiding targeted interventions across the “farm‐to‐fork” continuum. Ultimately, mitigating these dual threats requires coordinated action among regulatory authorities, the poultry industry and consumers to safeguard this vital protein source and preserve critical antimicrobials.

## 6 Conclusion

This study concludes that table eggs sold in the Rupandehi district of Nepal are a significant source of *Salmonella* and, more critically, a potent vehicle for the dissemination of MDR strains. The alarmingly high prevalence of resistance to ciprofloxacin, a first‐line antibiotic for treating severe salmonellosis, directly links antimicrobial use in local poultry production to a severe threat to human health. The evidence points unequivocally to the post‐farmgate supply chain as the primary stage for contamination amplification, highlighting critical failures in hygiene and temperature control at retail and restaurant levels. A coordinated One Health response is urgently needed, combining strict antimicrobial regulation, HACCP‐based food safety in the poultry industry, and public health campaigns on safe egg handling.

## Author Contributions

**Maadhabi Pokhrel**: investigation, methodology, writing – original draft, conceptualization. **Akash Adhikari**: writing – review and editing, formal analysis, data curation, methodology, visualization. **Sujan Adhikari**: writing – review and editing, formal analysis, data curation, validation. **Priyanka Bhatta**: investigation, data curation, project administration. **Samikshya Bhusal**: investigation, project administration, data curation. **Swagat Khanal**: writing – review and editing, software, visualization. **Abhishek Adhikari**: software, formal analysis, writing – review and editing. **Suman Kumar Singh**: conceptualization, methodology, validation, resources, supervision, writing – review and editing. **Birendra Shrestha**: conceptualization, methodology, supervision, project administration, writing – review and editing.

## Funding

The authors have nothing to report.

## Ethics Statement

This study did not involve experimental interventions on live animals. Egg samples were collected from commercial sources only. No personal information from farmers, retailers, or restaurant staff was collected; all data were anonymised.

## Consent

The authors have nothing to report.

## Conflicts of Interest

The authors declare no conflicts of interest.

## Data Availability

The datasets generated and analysed during the current study are available from the corresponding author upon reasonable request.
